# Association between genetic variants of *microRNA‐21* and *microRNA‐155* and systemic lupus erythematosus: A case‐control study from a Chinese population

**DOI:** 10.1002/jcla.24518

**Published:** 2022-06-16

**Authors:** Rong Wang, Anji Wei, Yingjie Zhang, Guidan Xu, Xuejuan Nong, Chunhong Liu, Yonglong Zeng, Huatuo Huang, Xiaoxia Pang, Wujun Wei, Chunfang Wang, Huayi Huang

**Affiliations:** ^1^ Department of Laboratory Medicine The Affiliated Hospital of Youjiang Medical University for Nationalities Baise China; ^2^ Mindray North America, 800 MacArthur Boulevard Mahwah New Jersey USA; ^3^ Department of Surgical Oncology Roswell Park Comprehensive Cancer Center, Elm and Carton Streets Buffalo New York USA

**Keywords:** genetic variants, *miR‐155*, *miR‐21*, systemic lupus erythematosus

## Abstract

**Background:**

Systemic lupus erythematosus (SLE) is a common autoimmune disease, and its pathogenesis remains unclear. The alteration of genetic materials is believed to play a role in SLE development. This study evaluated the association between the genetic variants of *microRNA‐21* (*miR‐21*) and *microRNA‐155* (*miR‐155*) and SLE.

**Methods:**

The SNaPshot genotyping method was used to detect the genotypes of selected SNPs in patients and controls. The expression of *miR‐21* and *miR‐155* was analyzed using reverse transcription‐quantitative polymerase chain reaction (RT‐qPCR). The functional annotation and the biological effects of SNPs were assessed by HaploReg V4.1 and Regulome DB V2.0 software. The Hardy–Weinberg equilibrium test was used to gather statistics, and odds ratios (ORs) and 95% confidence intervals (CIs) were evaluated by logistic regression.

**Results:**

The distribution difference of TA genotype in rs767649 was observed (TA vs. T/T: OR = 0.68, 95%CI, 0.48–0.95, *p* = 0.026). There was a significant difference in the T/A + A/A (T/A + A/A vs. T/T: OR = 0.68, 95%CI, 0.49–0.94, *p* = 0.020). A significant difference in T allele distribution was found in the depressed complement of SLE (T vs. A: OR = 0.67, 95%CI, 0.47–0.95, *p* = 0.026). There were significant differences in genetic variants of rs13137 between the positive and the negative SSB antibodies (Anti‐SSB) (T vs. A: OR = 0.67, 95%CI, 0.47–0.95, *p* = 0.026; T/A + T/T vs. AA: OR = 2.23, 1.18–4.49, *p* = 0.013). The expression levels of *miR‐21* and *miR‐155* were significantly higher in patients than in controls (*p* < 0.001).

**Conclusions:**

This study provides novel insight that genetic variants of rs767649 and rs13137 are associated with susceptibility to SLE.

## Graphical abstract

Association between genetic variants of rs767649 and rs13137 with systemic lupus erythematosus.

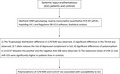

## 1 INTRODUCTION

Systemic lupus erythematosus (SLE) is one of the common systemic autoimmune diseases and causes multiple autoantibody production, immune complex, and disordered inflammatory cytokine.^1^, ^2^, ^3^ The prevalence of SLE currently ranges from 6.5 to 178.0 per 100,000 of the population, and SLE mainly affects females during the reproductive age.^4^ In the past several decades, the survival of SLE patients has improved due to innovative drugs, but the most suitable treatment remains insufficient. Immunosuppressants and biologicals have been used to modulate and manage the activity of SLE, keeping it in a remission clinical phase, preventing damage to organs and comorbidities, and are largely responsible for improved outcomes of SLE.^5^, ^6^ Traditionally, the complexity of genetic, hormonal, and environmental factors was associated with the diseases.^7^ With the advancement of research, however, accurate pathogenesis of the disease remains unknown, with many factors influencing the presentation of the disease. It is emphasized that genetic components play a role in the development of SLE. Genome‐wide association studies (GWASs) have authenticated many susceptible genes associated with SLE, underlining potential molecular mechanisms for developing the disease.^8^, ^9^, ^10^, ^11^ Nevertheless, abundant risk sites still need to be identified, making additional research necessary.

MicroRNAs (miRNAs) are small and non‐coding RNAs found in plants, animals, and humans. The mechanism for controlling gene expression was revealed in miRNAs, leading to negative regulation at the posttranscriptional level, usually by combining 3′‐ untranslated regions (3’‐UTR).^12^ Several studies have demonstrated that *microRNA‐21* (*miR‐21*) is involved in different kinds of diseases, such as cancers, diabetes, and neurological diseases.^13^, ^14^, ^15^, ^16^ Recently, some studies found that microRNAs were involved in different types of autoimmune diseases, including SLE.^17^, ^18^, ^19^, ^20^ The *miR‐21* is considered a prospective marker and has been identified in many autoimmune diseases.^21^ Some evidence has revealed that higher miR‐21 expression can be found in lupus when compared with control groups.^22^ Additionally, miR‐21 was overexpressed relative to wild‐type controls and connected with the severity of lupus in B6.Sle123 mice.^23^ *miR‐155* was able to modulate the immunologic development and responses in the pathogenesis of multiple sclerosis.^24^ *miR‐155* can be induced by inflammatory cytokines in innate immune cells.^25^ Others have found that the deletion of *miR‐155* reduces responses of autoantibody and relieves a lupus‐like disease in *Fas* ^***lpr***^ mice.^26^ Elevated levels of miR‐155 have also been found in human studies from patients with SLE.^27^ These emerging results illustrate that *miR‐21* and *miR‐155* could be crucial miRNAs in the occurrence and development of SLE.

In addition, several genes have been associated with the susceptibility of SLE, such as the *TNFAIP3* Gene and the Melatonin Pathway Gene.^28^, ^29^ Several studies have demonstrated that genetic variants in *miR‐21* and *miR‐155* are associated with disease susceptibility. Zhang J et al. described that rs1292037 is associated with the chemoresistance to cisplatin plus paclitaxel and the prognosis of patients with cervical cancer.^30^ Moreover, the T allele of rs13137 in the *miR‐21* offered protection against sepsis. The results suggest rs13137 is associated with the occurrence of sepsis.^31^ Assmann et al.^32^ indicate that rs767649 polymorphisms in *miR‐155* are associated with protection for Type 1 diabetes mellitus. A study that observed the Egyptian rheumatoid arthritis (RA) patients revealed that a functional variant of rs767649 may be an important site for the susceptibility of RA.^33^ Does a relationship between these SNPs and SLE exists? These observations prompted us to further investigate their role in SLE. For this hypothesis, we performed an association analysis in a cohort of Chinese patients with SLE.

## 2 METHODS

### 2.1 Research population

Two hundred ninety‐nine patients were enrolled in the study at the Affiliated Hospital of the Youjiang Medical University for Nationalities, Guangxi, China. Diagnosis of all SLE was performed according to the 1997 American College of Rheumatology classification (ACR) criteria for SLE and EULAR/ACR guidelines. The subjects included 240 females and 59 males, with 163 individuals collected between September 2016 and February 2019, a mean age of 38.18 ± 13.24 years, and mean age at onset of 39.81 ± 11.31 years. The following conditions were excluded: (1) patients with other autoimmune diseases; (2) patients with infection as co‐morbidity; (3) patients with severe heart, liver, and renal failure; (4) patients with malignant tumors, neurodegenerative diseases, and mental diseases; and (5) unwilling to participate in the investigation. Meanwhile, 298 healthy subjects from the same hospital who were coming for annual routine check‐ups were included. Those healthy subjects underwent physical examinations and several panels of laboratory testing with normal results. The study was conducted under the approval of the ethics committee of the Affiliated Hospital of Youjiang Medical University for Nationalities, and all participants provided consent.

### 2.2 Genotype analysis

The genomic DNA in the samples was extracted from blood samples using the standard procedures of a commercial DNA isolation kit (Tiangen, Beijing, China). PCR primers used in the study were designed by online primer 3.0 software (http://primer3.ut.ee/).^34^ The snapshot was used for genotyping analysis in all subjects. Related scripts and supporting data are stored at https://github.com/ronglearn/ *miR‐21* ‐and‐ *miR‐155* ‐SLE.

### 2.3 Detection of autoantibodies, complement, and analysis of microRNA expression

Anti‐DNA, anti‐SSA, anti‐SSB, anti‐Sm, and anti‐RNPP were analyzed by using the immunofluorescence method (Euroimmun,). The serum C3 and C4 complements were detected using a Roche Elecsys immunoturbidimetric assay on a Roche 702 chemistry analyzer platform (Roche Diagnostics,). Peripheral blood from a mononuclear cell was separated from subjects utilizing a separating medium according to the guideline (Meide Pacific biotechnology,). cDNA synthesis was performed using the Mir‐X miRNA First‐Strand Synthesis Kit (Takara,). Using the obtained cDNA as templates, miRNAs were then generated by qRT‐PCR using the ABI 7500 Real‐Time PCR system (Applied Biosystems,). U6 was used as an internal control.

### 2.4 Functional annotation and biological insights

The potential function annotation and biological effect of SNPs were assessed using HaploReg v4.1 (http://pubs.broadinstitute.org/mammals/haploreg/haploreg.php) ^35^ and RegulomeDB v2.0 (https://regulome.stanford.edu/regulome‐search).^36^

### 2.5 Statistical analysis

Genotype distributions were evaluated for departure from the Hardy–Weinberg equilibrium (HWE) test by the goodness‐of‐fit χ^2^. The categorical variables are expressed in absolute number and percentage, and the continuous variables are expressed as mean ± standard deviation (normal/parametric distribution) or as the median and interquartile range (25%–75%) (no normal/no parametric distribution). The Kolmogorov–Smirnov test was used to assess the normality of data distribution. Then the Mann–Whitney test was used for the analysis of non‐parametric data. The allelic and genotype frequencies were calculated by direct count. Significant differences in the genetic variant between cases and controls were analyzed by the chi‐square test. Odds ratios (ORs) and 95% confidence intervals (95%CIs) were evaluated using logistic regression under age and sex. Estimation of Haplotypes was performed using SHEsis software (http://analysis.bio‐x.cn/myAnalysis.php).^37^ Two‐tailed *p* values <0.05 were considered statistically significant. Statistical analysis in the study was performed by the SPSS software (version 23.0).

## 3 RESULTS

### 3.1 Basic characteristics of subjects

Baseline characteristics of the two groups in the study are shown in (Table S1). There were no significant differences in age and gender between patients and controls, respectively (*p* = 0.107, *p* = 0.226). Moreover, the other clinical characteristics of patients are displayed in (Table S2).

### 3.2 Association of rs767649 and rs13137 genetic variant and SLE

Genotype frequencies of the SNPs were coincident with the Hardy–Weinberg Equilibrium. The frequencies of genotypes and alleles in the SNPs are shown in Table 1. A significant difference in the TA genotype distribution in rs767649 compared with the T/T genotype between patients and controls was observed (T/A vs. T/T: OR = 0.68, 95%CI, 0.48–0.95, *p* = 0.026). Based on the evaluation of the dominant model and recessive model, there is a significant difference in the dominant model of rs767649 (T/A + A/A vs. T/T: OR = 0.68, 95%CI, 0.49–0.94, *p* = 0.020). In addition, a significant difference was observed in the A allele (T/A + A/A vs. T/T: OR = 0.77, 95%CI, 0.60–0.99, *p* = 0.038).

**TABLE 1:** Distribution of genotypes in rs767649 and rs13137 gene between SLE and controls

Genetic variant	SLE (%)	Controls (%)	OR (95%CI)	Adjusted OR (95%CI)^*^	*p*	Adjusted *p* ^*^
rs767649						
TT	158 (52.8)	129 (43.3)	1.00 (Ref)	1.00 (Ref)	0.026	0.026
TA	115 (39.5)	138 (46.3)	0.68 (0.48–0.96)	0.68 (0.48–0.95)		
AA	26 (8.7)	31 (10.4)	0.69 (0.39–1.21)	0.70 (0.39–1.23)	0.192	0.213
Dominant						
TT	158 (52.8)	129 (43.3)	1.00 (Ref)	1.00 (Ref)	0.019	0.020
TA + AA	141 (47.2)	169 (56.7)	0.68 (0.49–0.94)	0.68 (0.49–0.94)		
Recessive						
AA	26 (8.7)	31 (10.4)	1.00 (Ref)	1.00 (Ref)	0.478	0.513
TA + TT	273 (91.3)	267 (89.6)	1.22 (0.71–2.11)	1.20 (0.69–2.09)		
T	43 (72.1)	396 (66.4)	1.00 (Ref)	1.00 (Ref)	0.035	0.038
A	167 (27.9)	200 (33.6)	0.77 (0.60–0.98)	0.77 (0.60–0.99)		
rs13137						
AA	104 (34.8)	100 (33.5)	1.00 (Ref)	1.00 (Ref)	0.655	0.682
AT	136 (45.5)	142 (47.7)	0.92 (0.64–1.32)	0.93 (0.65–1.33)		
TT	59 (19.7)	56 (18.8)	1.01 (0.64–1.60)	1.03 (0.65–1.63)	0.956	0.909
Dominant						
AA	104 (34.8)	100 (33.5)	1.00 (Ref)	1.00 (Ref)	0.752	0.792
TA + TT	195 (65.2)	198 (66.5)	0.95 (0.68–1.33)	0.96 (0.68–1.34)		
Recessive						
TT	59 (19.7)	56 (18.8)	1.00 (Ref)	1.00 (Ref)	0.771	0.735
TA + AA	240 (80.3)	242 (81.2)	0.94 (0.63–1.41)	0.93 (0.62–1.40)		
A	344 (57.5)	342 (57.4)	1.00 (Ref)	1.00 (Ref)	0.960	0.991
T	254 (42.5)	254 (42.6)	1.01 (0.80–1.27)	1.00 (0.79–1.26)		

Abbreviations: OR, odds ratio; 95% CI, 95% confidence interval; Ref, reference.

*Adjusted by age and sex.

### 3.3 Haplotypes analysis of selected SNPs

Firstly, the establishment of haplotypes in the selected SNPs was implemented with SHEsis software. Afterwards, the data was analyzed using the above statistical tool, and the results are described in Table 2. Finally, four haplotypes were established between the SLE and controls. No significant differences appeared in the haplotypes compared to the referenced maximum haplotypes (*p* > 0.05).

**TABLE 2:** Haplotype analysis of the SNPs in SLE patients and controls

Haplotypes	SLE	Controls	OR (95%)	*p*
AT	255	239	1.00 (Ref)	
AA	90	103	0.82 (0.59–1.14)	0.240
TA	77	97	0.74 (0.53–1.05)	0.095
TT	176	157	1.05 (0.80–1.39)	0.728

Abbreviations: OR, odds ratio; 95% CI, 95% confidence interval; Ref, reference.

### 3.4 Association between SNPs and expression of miRNAs

**FIGURE 1:**
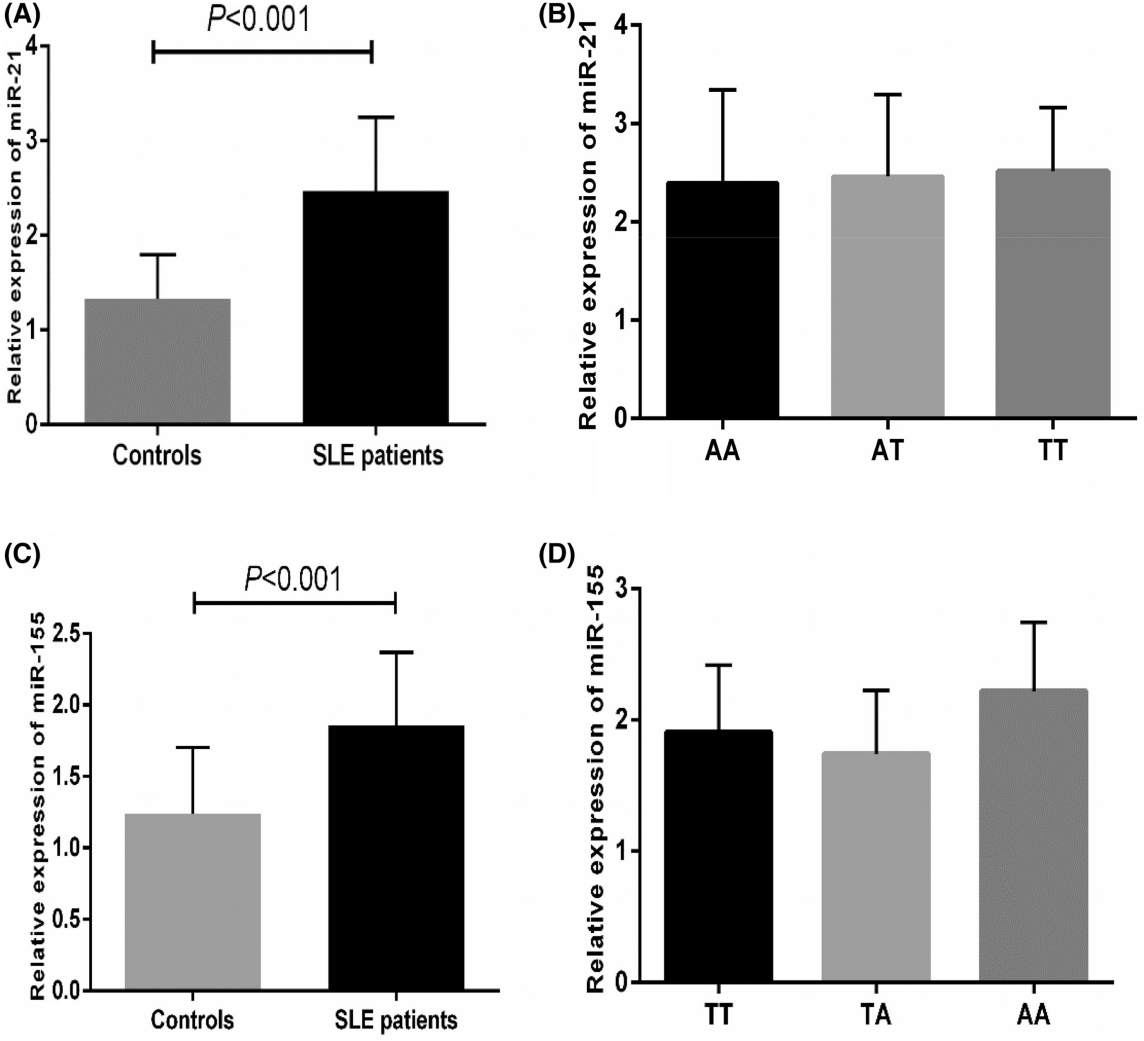
Relative expression levels of miR‐21 and miR‐155. A. Relative expression level of miR‐21 in SLE patients and controls. B. Relative expression level of miR‐21 between different genotypes in rs13137. C. Relative expression level of miR‐21 between different genotypes in rs767649. Expression levels of *miR‐21* and *miR‐155*. A, Expression levels of *miR‐21* in patients with SLE and controls. B, Expression levels of *miR‐21* in genotypes of rs767649 from SLE patients. C, Expression levels of *miR‐155* in patients with SLE and controls. D, Expression levels of *miR‐155* in genotypes of rs13137 from SLE patients

Expression levels of *miR‐21* and *miR‐155* were significantly higher in patients with SLE compared to controls (*p* < 0.01, Figure 1A,C). To explore if the genotypes of the SNPs affected the levels of miR‐21 and miR‐155, the relationship between the genotypes and expression of the miRNAs was analyzed. However, no significant difference was found in the study (*p* > 0.05, Figure 1B,D).

### 3.5 Association between SNPs and disease characteristics

In order to investigate the potential association of the genotypes and disease characteristics, we evaluated the data and summarized the results in Table 3 and Table 4. Consequently, we found that the T allele distribution was related to depressed complement in SLE (T vs. A: OR = 0.67, 95%CI, 0.47–0.95, *p* = 0.026), Table 5. There are significant differences in genotype frequency and allele frequency in rs13137 between the positive and the negative Anti‐SSB (T vs. A: OR = 0.67, 95%CI, 0.47–0.95, *p* = 0.026; T/A + T/T vs. A/A: OR = 2.23, 1.18–4.49, *p* = 0.013), Table 6.

**TABLE 3:** Association of rs767649 genetic variant with clinical manifestations

Clinical features	+/−	Allele [*n*]		*p*	Adjusted *p* ^*^	Genotype [*n*]		*p*	Adjusted *p* ^*^
Malar rash		T	A			TT	TA + AA		
	+	120	54	0.278	0.254	44	43	0.615	0.544
	−	311	113			114	98		
Photosensitivity	+	244	92	0.736	0.738	91	77	0.604	0.536
	−	187	75			67	64		
Leucopenia	+	270	96	0.245	0.240	104	79	0.083	0.078
	−	161	71			54	62		
Anemia	+	228	98	0.203	0.228	81	82	0.232	0.306
	−	203	69			77	59		
Depressed complement	+	300	120	0.589	0.613	108	102	0.425	0.474
	−	131	47			50	39		
Renal disorder	+	229	97	0.275	0.262	81	82	0.232	0.217
	−	202	70			77	59		
Neurologic disorder	+	96	36	0.850	0.897	34	32	0.807	0.725
	−	335	131			124	109		
Arthritis	+	261	99	0.775	0.830	97	83	0.656	0.734
	−	170	68			61	58		
Anti‐dsDNA	+	216	82	0.824	0.812	80	69	0.770	0.722
	−	215	85			78	72		
Anti‐RNP	+	170	70	0.580	0.589	62	58	0.739	0.752
	−	261	97			96	83		
Anti‐Sm	+	179	67	0.753	0.774	65	58	0.999	0.963
	−	252	100			93	83		
Anti‐SSA	+	288	114	0.736	0.766	104	97	0.585	0.629
	−	143	53			54	44		
Anti‐SSB	+	96	40	0.660	0.655	35	33	0.796	0.804
	−	335	127			123	108		

Abbreviations: OR, odds ratio. 95% CI, 95% confidence interval; Ref, reference.

*Adjusted by age and sex.

**TABLE 4:** Association of rs13137 genetic variant with clinical manifestations

Clinical features	+/−	Allele [*n*]		*p*	Adjusted *p* ^*^	Genotype [*n*]		*p*	Adjusted *p* ^*^
		A	T			AA	TA + TT		
Malar rash	+	99	75	0.842	0.885	30	57	0.944	0.963
	−	245	179			74	138		
Photosensitivity	+	190	146	0.584	0.534	58	110	0.915	0.871
	−	154	108			46	85		
Leucopenia	+	222	144	0.052	0.062	70	113	0.114	0.116
	−	122	110			34	82		
Anemia	+	197	129	0.116	0.139	62	101	0.247	0.210
	−	147	125			42	94		
Depressed complement	+	254	166	0.025	0.026	80	130	0.065	0.065
	−	90	88			24	65		
Renal disorder	+	197	129	0.116	0.111	61	102	0.294	0.293
	−	147	125			43	93		
Neurologic disorder	+	80	52	0.417	0.386	25	41	0.550	0.535
	−	264	202			79	154		
Arthritis	+	201	159	0.303	0.330	59	45	0.371	0.373
	−	143	95			121	74		
Anti‐dsDNA	+	179	119	0.210	0.225	57	92	0.209	0.218
	−	165	135			47	103		
Anti‐RNP	+	136	104	0.728	0.720	43	77	0.755	0.756
	−	208	150			61	118		
Anti‐Sm	+	138	108	0.555	0.572	39	84	0.351	0.354
	−	206	146			65	111		
Anti‐SSA	+	226	176	0.335	0.336	70	131	0.982	0.990
	−	118	78			34	64		
Anti‐SSB	+	68	68	0.043	0.043	15	53	0.012	0.013
	−	276	186			89	142		

Abbreviations: OR, odds ratio; 95% CI, 95% confidence interval; Ref, reference.

*Adjusted by age and sex.

**TABLE 5:** Association of rs13137 genetic variant with depressed complement in SLE patients

Allele	Depressed complement		OR (95%CI)	Adjusted OR (95%CI)^*^	*p*	Adjusted *p* ^*^
	+	−				
A	25	90	1.00 (Ref)	1.00 (Ref)	0.025	0.026
T	166	88	0.67 (0.47–0.95)	0.67 (0.47–0.95)		

Abbreviations: OR, odds ratio; 95% CI, 95% confidence interval; Ref, reference.

*Adjusted by age and sex.

**TABLE 6:** Association of rs13137 genetic variant with anti‐SSB in SLE patients

Allele	Anti‐SSB		OR (95%CI)	Adjusted OR (95%CI)^*^	*p*	Adjusted *p* ^*^
	+	−				
A	68	276	1.00 (Ref)	1.00 (Ref)	0.043	0.043
T	68	186	1.48 (1.01–2.18)	1.49 (1.01–2.19)		
Genotype						
AA	15	89	1.00 (Ref)	1.00 (Ref)	0.012	0.013
TA + TT	53	142	2.22 (1.18–4.16)	2.23 (1.18–4.49)		

Abbreviations: OR, odds ratio; 95% CI, 95% confidence interval; Ref, reference.

*Adjusted by age and sex.

### 3.6 Functional annotation and biological insights

RegulomeDB and HaploReg are online databases that can better annotate the function of SNPs. The regulatory role of SNPs was annotated in HaploReg v4.1 and Regulome DB v2.0. Based on HaploReg v4.1, we found that the region around rs767649 mainly enriched on gene promoters and enhancers marked by H3K4me1, H3K4me3, and H3K27ac in some immune cells, such as T cell, B cell, monocytes, and skin. rs13137 significantly enriched on gene promoters and enhancers marked by H3K4me1, H3K4me3, H3K27ac, and H3K9ac in the above cells and skins. From Regulome DB v2.0, rs767649 and rs13137 had Regulome DB scores of 0.36261 and 0.60906, respectively. If Regulome DB scores <3, it means that SNPs had a relatively high degree of evidence for potential regulatory function.

## 4 DISCUSSION

To date, past studies accurately illustrate that miRNAs play an essential role in human immune homeostasis, imbalance in the immune‐cell development, and functions related to autoimmune diseases. Importantly, the role of genetic variants in miRNAs has been studied in other diseases, including little‐known autoimmune diseases.^38^, ^39^ Polymorphisms affecting miRNA expression may represent a vital risk factor in disease susceptibility.^40^ We found the T/A genotype, T/A + A/A genotypes, and A allele of rs767649 gene were associated with protection from SLE. Additionally, different distribution of T allele in rs13137 was associated with depressed complement as well as significant differences in genotype frequency and allele frequency in the classification of Anti‐SSB.

The human *miR‐21* gene is mapped to chromosome 17q23.2, and the *miR21* (rs13137) genetic variant consists of T > A substitution. miR‐21 functions as an anti‐apoptotic and pro‐survival factor. Furthermore, recent studies have reported that miRNAs in the body can be tested in circulation and become potential biomarkers in various diseases. Alteration in miR‐21 expression has been observed in some autoimmune diseases.^41^, ^42^ miRNA profile and RT‐qPCR were performed to estimate the abnormality expressed in circulating miRNAs in individuals with SLE compared to patients with RA and also to healthy controls. Wang H et al.^43^ showed that miR‐21 was upregulated in the SLE patients and was also significantly increased in RA patients. Another study tested the levels of miR‐31, miR‐21, and IL‐2 concentrations in the plasma of SLE patients. They found that miR‐21 was upregulated and negatively correlated with IL‐2 levels.^44^, ^45^ One study also found that miR‐21 was elevated in the peripheral blood mononuclear cells in lupus patients,^46^ and expression of miR‐155 could be a potential biomarker of SLE diagnosis and disease activity prediction.^47^

For treatment, there were some attempts to use microRNA as a means of therapeutic targeting for SLE.^48^, ^49^, ^50^ In an in vivo study, silencing of miR‐21 under a tiny seed‐targeting locked‐nucleic acid (LNA) reversed splenomegaly resulted in approximately 20% de‐repression of programmed cell death 4 (PDCD4) in naïve CD4+ T cells and recovery of lupus mice.^23^ An examination of miRNA expression profiles in patients with SLE found that miR‐21 and miR‐155 were overexpressed in peripheral blood mononuclear cells from the SLE group.^46^ Consistent with these discoveries, our results observed that miR‐21 and miR‐155 were upregulated in peripheral blood mononuclear cells in SLE patients.

The rs13137 T allele of the *miR‐21* gene in a carrier was 0.755 times less likely to be infected with sepsis compared to the A allele carrier. The T allele of the rs13137 was found to be a protective factor against sepsis.^31^ In our study, we also found that the T allele distribution was associated with depressed complement in SLE and significant differences in genotype frequency and allele frequency in rs13137 between the positive and the negative Anti‐SSB. *miR‐21* inhibits the polarization of the immune response towards Th1 cells and promotes the Th2 response; the A allele could promote the Th2 response, and the T allele could promote the Th1 response. Therefore, this shift of the Th2 toward Th1 could illustrate the lower chance of decreased levels of complement and anti‐SSB antibodies among those carrying the T allele of this variant ^51^, ^52^, ^53^, ^54^, ^55^, ^56^, ^57^, ^58^.

MicroRNA‐155 (miRNA‐155) is encoded by the human B‐cell integration cluster gene, and the *miR155* T > A (rs767649) genetic variant consists of T > A substitution. A study in RA patients detected that *miR‐155* and rs767649 may play an important role in the increased risk of RA, stressing *miR‐155* as a therapeutic target in the treatment of RA.^33^ The A allele of rs767649 was independently associated with an increased risk of diabetic retinopathy.^59^ Ji et al believed that the minor allele of rs767649 in the promoter was significantly associated with an increased risk of hepatocellular carcinoma. The T/T genotype was significantly associated with the 1.94‐fold poor survival cancer.^60^ However, SNP was found to play a protective role against other diseases. rs767649 genetic variants were related to protection from Type 1 diabetes mellitus, and the strongest association was observed for the dominant model.^32^ Results showed that the rs767649 T/T genotype was related to a significantly reduced risk for cervical cancer.^61^ Similarly, we also observed that the rs767649 gene in a protective role was associated with SLE. On the other hand, in regards to the possible inhibitory role of the *miR‐155* in the immune response, patients with the A allele of the *miR155* (rs767649) may have more inhibitory effect on immune response than those carrying the T allele, which may explain the potential protective effect of the A allele (T/A + A/A vs T/T) on SLE susceptibility. The difference in frequency distribution in SNPs should be explained in different diseases.

Greater sample sizes for the association of SNPs will be useful to understand their roles in diseases.^39^ Several limitations need to be discussed in our study. Firstly, we only selected some important SNP sites to investigate the association between the SNP and SLE. Studies targeting more SNP sites in the *miR‐21* gene and *miR‐155* gene will be helpful to understand their roles in the disease. Secondly, due to the limited information, we were unable to evaluate the dose‐dependence between SNPs and antibodies. Thirdly, a larger sample size will be necessary to investigate their association.

## 5 CONCLUSIONS

In conclusion, this study suggests that rs13137 and rs767649 may contribute to SLE susceptibility and clinical features. However, designed studies with different populations and evaluations of functional mechanisms in vitro and in vivo will be worth conducting to confirm these findings.

## AUTHOR CONTRIBUTIONS

Wang R wrote the draft. Wei A, Zhang Y, Xu G, Liu C, and Nong X involved in methodology and investigation. Zeng Y, Huang H^a^, and Lei M involved in investigation, review and editing. Pang X and Wei W formally analyzed the data. Wang C involved in conceptualization, funding acquisition, and played as a principal investigator. Huang H^b^ edited the manuscript and involved in critical discussion.

## CONFLICT OF INTEREST

The authors declare that they have no known competing financial interests or personal relationships that could have appeared to influence the work reported in this paper.

## Supporting information


Appendix S1

## Data Availability

The datasets generated and/or analyzed during the current study are available from the corresponding author on reasonable request. Data available on request due to privacy/ethical restrictions
