# Supplementary material for: Association between genetic variants of microRNA‐21 and microRNA‐155 and systemic lupus erythematosus: A case‐control study from a Chinese population
Source: J Clin Lab Anal. 2022 Jun 16;36(7):e24518. doi: 10.1002/jcla.24518 (PMC9279951; doi:10.1002/jcla.24518)
Supplement: Supplementary file 1 — Appendix S1 [file JCLA-36-e24518-s001.docx]

**Supplementary Table S1 Clinical manifestations of SLE patients and controls**

| Characteristics | SLE (%) | Controls (%) | *P* |
| --- | --- | --- | --- |
| Age (years) | 38.18±13.24 | 39.81±11.31 | 0.107 |
| Male/Female | 59/240 | 71/227 | 0.226 |
| Malar rash | 87 (29.1) | - | - |
| Photosensitivity | 168 (56.2) | - | - |
| Leucopenia | 183 (61.5) | - | - |
| Anemia | 163 (54.5) | - | - |
| Complement depressed | 210 (70.2) | - | - |
| Renal disorder | 163 (54.5) | - | - |
| Neurologic disorder | 66 (22.1) | - | - |
| Arthritis | 180 (60.2) | - | - |
| Anti-dsDNA | 149 (49.8) | - | - |
| Anti-RNP | 120 (40.1) | - | - |
| Anti-Sm | 123 (41.1) | - | - |
| Anti-SSA | 201 (67.2) | - | - |
| Anti-SSB | 68 (22.7) | - | - |
| Note: SLE, systemic lupus erythematosus. Anti-dsDNA, anti-double strand DNA antibody. Anti-RNP, anti-ribonuclear protein antibody. Anti-Sm, anti-Smith. Anti-SSA, anti-SjÖgren syndrome antigen A antibody. Anti-SSB, anti-SjÖgren syndrome antigen B antibody. | | | |

**Supplementary Table S2 Distribution of alleles in the rs767649 and rs13137 between SLE patients and controls**

| Polymorphisms | SLE (%) | Controls (%) | OR (95%CI) | Adjusted  OR (95%CI) ^*^ | *P* | Adjusted *P*^*^ |
| --- | --- | --- | --- | --- | --- | --- |
| rs767649 |  |  |  |  |  |  |
| T | 43(72.1) | 396(66.4) | 1.00 (Ref) | 1.00 (Ref) |  |  |
| A | 167(27.9) | 200(33.6) | 0.77(0.60-0.98) | 0.77(0.60-0.99) | 0.035 | 0.038 |
| rs13137 |  |  |  |  |  |  |
| A | 344(57.5) | 342(57.4) | 1.00 (Ref) | 1.00 (Ref) |  |  |
| T | 254(42.5) | 254(42.6) | 1.01(0.80-1.27) | 1.00(0.79-1.26) | 0.960 | 0.991 |
| Note: SLE, systemic lupus erythematosus. OR, odds ratio. 95% CI, 95% confidence interval. Ref, reference. *Adjusted by age and gender. | | | | | | |
